# Supplementary material for: Entrainment and maintenance of an internal metronome in supplementary motor area
Source: eLife. 2018 Oct 22;7:e38983. doi: 10.7554/eLife.38983 (PMC6249004; doi:10.7554/eLife.38983)
Supplement: Transparent reporting form [file elife-38983-transrepform.pdf]

eLIFE
1st Floor
24 Hills Road
Cambridge CB2 1JP, UK
P 01223 855340
W elifesciences.org
T @elife

eLife's transparent reporting form

We encourage authors to provide detailed information *within their submission* to facilitate the interpretation and replication of experiments. Authors can upload supporting documentation to indicate the use of appropriate reporting guidelines for health-related research (see EQUATOR Network), life science research (see the BioSharing Information Resource), or the ARRIVE guidelines for reporting work involving animal research. Where applicable, authors should refer to any relevant reporting standards documents in this form.

If you have any questions, please consult our Journal Policies and/or contact us: editorial@elifesciences.org.

Sample-size estimation

- You should state whether an appropriate sample size was computed when the study was being designed
- You should state the statistical method of sample size computation and any required assumptions
- If no explicit power analysis was used, you should describe how you decided what sample (replicate) size (number) to use

Please outline where this information can be found within the submission (e.g., sections or figure legends), or explain why this information doesn't apply to your submission:

-We recorded from 2 rhesus macaques which is the minimum acceptable number of replicates in the field of awake monkey electrophysiology. This information can be found in the Results section, page 8.

-We performed 84 and 47 recording sessions in monkeys 1 and 2, respectively. On each recording session monkeys performed 800-1100 trials. We did not perform a power analysis. The number of recording sessions is limited by a variety of experimental factors such as the dura matter integrity in the recording site. The number of trials per session is largely determined by the attention span and reward satiety of each monkey.

Replicates

- You should report how often each experiment was performed
- You should include a definition of biological versus technical replication
- The data obtained should be provided and sufficient information should be provided to indicate the number of independent biological and/or technical replicates
- If you encountered any outliers, you should describe how these were handled
- Criteria for exclusion/inclusion of data should be clearly stated
- High-throughput sequence data should be uploaded before submission, with a private link for reviewers provided (these are available from both GEO and ArrayExpress)

Please outline where this information can be found within the submission (e.g., sections or figure legends), or explain why this information doesn't apply to your submission:

eLife Sciences Publications, Ltd is a limited liability non-profit non-stock corporation incorporated in the State of Delaware, USA, with company number 5030732, and is registered in the UK with company number FC030576 and branch number BR015634 at the address 1st Floor, 24 Hills Road, Cambridge CB2 1JP | August 2014

eLIFE
1st Floor
24 Hills Road
Cambridge CB2 1JP, UK
P 01223 855340
W elifesciences.org
T @elife

-We state the number of monkeys and recording replicates in the Results section, page 8 (biological replicates).

-In Methods section, page 37 we provide the number of technical replicates in the analysis of correct and error trials by a logistic classifier.

Statistical reporting

- Statistical analysis methods should be described and justified
- Raw data should be presented in figures whenever informative to do so (typically when N per group is less than 10)
- For each experiment, you should identify the statistical tests used, exact values of N, definitions of center, methods of multiple test correction, and dispersion and precision measures (e.g., mean, median, SD, SEM, confidence intervals; and, for the major substantive results, a measure of effect size (e.g., Pearson's r, Cohen's d)
- Report exact p-values wherever possible alongside the summary statistics and 95% confidence intervals. These should be reported for all key questions and not only when the p-value is less than 0.05.

Please outline where this information can be found within the submission (e.g., sections or figure legends), or explain why this information doesn't apply to your submission:

-Statistical tests, central tendency (standard error) and dispersion estimates (IQR) are described in the Results (page 4) and Methods sections (page 35) of the manuscript. For convenience, at the end of this document we provide a table with each statistical test, significance, and parameters.

(For large datasets, or papers with a very large number of statistical tests, you may upload a single table file with tests, Ns, etc., with reference to sections in the manuscript.)

Group allocation

- Indicate how samples were allocated into experimental groups (in the case of clinical studies, please specify allocation to treatment method); if randomization was used, please also state if restricted randomization was applied
- Indicate if masking was used during group allocation, data collection and/or data analysis

Please outline where this information can be found within the submission (e.g., sections or figure legends), or explain why this information doesn't apply to your submission:

We did not perform analyses by group. Data from both monkeys was pooled and analysed together. We state this in Results section, page 8, line 145.

Additional data files ("source data")

- We encourage you to upload relevant additional data files, such as numerical data that are represented as a graph in a figure, or as a summary table
- Where provided, these should be in the most useful format, and they can be uploaded as "Source data" files linked to a main figure or table
- Include model definition files including the full list of parameters used

eLife Sciences Publications, Ltd is a limited liability non-profit non-stock corporation incorporated in the State of Delaware, USA, with company number 5030732, and is registered in the UK with company number FC030576 and branch number BR015634 at the address 1st Floor, 24 Hills Road, Cambridge CB2 1JP | August 2014

- Include code used for data analysis (e.g., R, MatLab)
- Avoid stating that data files are "available upon request"

Please indicate the figures or tables for which source data files have been provided:

-We made use of the Chronux Matlab toolbox for spectral analyses. We state this in the Methods section on page 36, line 671.

Statistical tests

Description	Test	Parameters	p values/Explained variance	Location	Additional notes
Regression between elapsed time and reaction time	Linear regression	Linear fit= Y=A*x+B, Estimated parameters: A, B	R squared=.72	Fig. 1C	
Comparison between pre-burst amplitude and burst amplitude	Wilcoxon paired samples test	n=939, T=2123, Z=24.9443	p<.000001	Fig. 2D	
Regression between elapsed time and Gamma amplitude in maintenance period	Non-linear regression	Exponential fit: y= A*exp(B*x)+C, Estimated parameters: A, B, C	R squared=.8667	Fig. 3C	
Chi-square tests between distributions of bursts onsets (between consecutive intervals), Distribution: 10 bins	Chi-square	102.31, 132.36, 69.63, 181.77, 320.97, 606.78	p<.001 (Bonferroni corrected)	Fig. 6B	Chi-square values, interval duration: 500, 750, 1000 ms
Regression between elapsed time and Gamma amplitude in entrainment period	Linear regression	Linear fit= Y=A*x+B, Estimated parameters: A, B	R squared=.94	Fig. 6D	
STA Power: Interaction between condition (Baseline, Entrainment, Maintenance) and Band (Alpha, Beta, Gamma)	Factorial ANOVA: Interaction Condition*Band	Interaction: SS=000029, DF=4, MS=0.000007 F=12.112	p<.000001	Fig. 8B	See methods
	Repeated measure: Condition	Error: SS=000407, DF=672, MS=0.000001			
Posthoc comparisons between Gamma power in entrainment and maintenance vs. Baseline	Bonferroni test	Comparison: Gamma power in Maintenance vs. Baseline	p=.000001	Fig. 8B	See methods
		Comparison: Gamma power in Entrainment vs. Baseline	p=.037		
Comparison between STA power at Gamma range around switch time and halfway between switch	t-test, dependent samples	n=113, t=-4.36352	p=.000029	Fig. 8C	Mean amplitude between 30-40 Hz.
Comparison between STA power at Gamma range around switch time and halfway between switch (Jittered data)	t-test, dependent samples	n=113, t=-1.25	p=.21	Fig. 8C	Gray traces Fig. 8C
Coherency of Gamma power between simultaneous recording sites	Linear regression	Linear fit= Y=A*x+B, Estimated parameters: A, B	R squared=.7284	Fig. 8D	See methods
